# Alcohol consumption, smoking, and weight loss outcomes: findings from a 12-month digital lifestyle intervention

**DOI:** 10.1093/eurpub/ckag072

**Published:** 2026-05-19

**Authors:** Emma R Wu, Anu Joki, Mikko S Venäläinen, Laura-Unnukka Suojanen, Kirsi H Pietiläinen, Aila J Ahola

**Affiliations:** University of South Florida Morsani College of Medicine, Tampa, FL, United States; Obesity Research Unit, Research Program for Clinical and Molecular Metabolism, Faculty of Medicine, University of Helsinki, Helsinki, Finland; Healthy Weight Hub, Abdominal Centre, Helsinki University Hospital and University of Helsinki, Helsinki, Finland; Department of Medical Physics, Turku University Hospital and University of Turku, Turku, Finland; Healthy Weight Hub, Abdominal Centre, Helsinki University Hospital and University of Helsinki, Helsinki, Finland; Obesity Research Unit, Research Program for Clinical and Molecular Metabolism, Faculty of Medicine, University of Helsinki, Helsinki, Finland; Healthy Weight Hub, Abdominal Centre, Helsinki University Hospital and University of Helsinki, Helsinki, Finland; Obesity Research Unit, Research Program for Clinical and Molecular Metabolism, Faculty of Medicine, University of Helsinki, Helsinki, Finland; Healthy Weight Hub, Abdominal Centre, Helsinki University Hospital and University of Helsinki, Helsinki, Finland

## Abstract

To investigate associations between alcohol consumption, smoking, and weight loss outcomes over a 12-month digital lifestyle intervention, the Healthy Weight Coaching. Weight, height, and waist circumference were self-reported at baseline, followed by weekly weight and quarterly waist circumference reporting. Interpolated weights were used to calculate body mass index at 3, 6, 9, and 12 months. At these timepoints, relative changes from baseline in weight and waist circumference were calculated. On web-based questionnaires, participants reported alcohol consumption (frequency, single-session dose) and smoking (status, daily dose, start age, end year). Participants were categorized as abstinent, low-risk drinkers, and risky drinkers (men: >14 units/week or >6 units/occasion; women: >7 units/week or >5 units/occasion), and as current, former and non-smokers. At baseline, of the 1719 participants (83.3% women, median age 51 years, body mass index 39.1 kg/m^2^), 20.9% reported abstinence and 16.2% risky drinking, while 12.1% and 22.3% were current and former smokers, respectively. Alcohol consumption decreased over the program, driven by reductions among baseline risky drinkers. Among baseline non-drinkers, increased alcohol intake over 12 months was linked to smaller waist circumference reduction (weekly dose, *B* = 0.762, *P* = 0.005; single-session dose, *B* = 1.168, *P* = 0.020). Instead, among baseline risky drinkers, cutting alcohol intake was linked to greater weight loss (weekly dose, *B* = 0.062, *P* = 0.034; single-session dose, *B* = 0.321, *P* < 0.001), and larger waist circumference reduction (single-session dose, *B* = 0.381, *P* = 0.031). Higher number of pack-years was associated with attenuated waist circumference reduction (*B* = 0.059, *P* = 0.002). Addressing alcohol consumption and smoking may improve weight loss outcomes in digital lifestyle interventions. The trial is registered at clinicaltrials.gov (Clinical Trials Identifier NCT04019249).

## Introduction

The global prevalence of obesity has increased substantially over recent decades [1], raising significant concern regarding its long-term implications for public health. Obesity is a well-established risk factor for cardiovascular disease, type 2 diabetes, several forms of cancer, and other chronic conditions [2]. Encouragingly, even a modest 5% reduction in body weight can lead to clinically meaningful improvements in multiple health markers [3], highlighting the importance of elucidating the determinants of successful weight loss.

Alcohol consumption and smoking both influence weight regulation [4, 5]. Light-to-moderate alcohol consumption appears weight-neutral, whereas heavy drinking contributes to excess energy load and impairs lipid oxidation, promoting visceral fat accumulation and cardiometabolic risk [5–7]. Nicotine can suppress appetite and increase resting energy expenditure, explaining why smokers often have lower body weight than non-smokers [8]. Paradoxically, heavy smoking is associated with greater obesity risk, potentially due to adverse metabolic effects, reduced physical activity, or unhealthy dietary patterns [4]. Smoking cessation frequently results in weight gain, with the magnitude increasing with duration of prior smoking [4].

Traditionally, behavioral weight loss programs have focused on diet and physical activity, with counselling on alcohol use and smoking less commonly included or systematically evaluated [9]. Evidence suggests that heavy alcohol use is associated with suboptimal long-term weight loss [10], and moderate consumption may attenuate some metabolic benefits of weight loss [11]. Findings on smoking have been mixed, with studies showing both greater [12] and poorer weight loss among current smokers [13].

Given these complex associations, it is important to consider their relevance in real-life lifestyle interventions. Most research has been conducted in face-to-face programs, while evidence from digital lifestyle interventions remains limited. Digital programs offer scalable, cost-efficient obesity treatment with efficacy comparable to face-to-face methods [14, 15]. However, the roles of alcohol and smoking in shaping individual outcomes within digital interventions remain insufficiently understood. Because digital interventions rely on self-monitoring and autonomous behavior change, examining these associations in real-world programs may provide additional insights into factors shaping weight loss outcomes. Using data from a 12-month structured digital lifestyle intervention, Healthy Weight Coaching (HWC), our primary aims were to assess if program participation was associated with reduced alcohol consumption, and if smoking habits or changes in alcohol consumption predicted weight loss outcomes. We hypothesized that alcohol consumption would decline among users and that this reduction would support weight loss. For smoking, we hypothesized that a greater number of pack-years, reflecting cumulative exposure, would impair weight loss success. As secondary aims, we examined baseline associations of alcohol consumption and smoking with anthropometric measures, including body mass index (BMI) and waist circumference, and the clustering of these habits in relation to weight loss outcomes.

## Methods

The HWC is an ongoing, referral-based, 12-month digital lifestyle intervention developed at Helsinki University Hospital as part of routine obesity care in Finland. It is delivered through the HealthyWeightHub.fi platform within the nationwide Health Village service [16]. The automated program includes weekly one-hour modules covering lifestyle and behavioral change topics, including nutrition, physical activity, rest, and psychological well-being. In addition to the automated modules, each participant is assigned a health-care professional as a personal digital coach who provides support according to a standardized protocol while tailoring guidance to individual needs. The program targets adults with overweight or obesity willing to engage in digital treatment. For these analyses, we considered data from patients enrolled between October 2016 and September 2020, who completed a two-week trial period. Participants were excluded if they used other weight loss methods beyond HWC (bariatric surgery, gastric balloon, obesity medication, or very low-energy diet) or lacked data on alcohol consumption and smoking. Apart from age and sex obtained from a national registry, all data were self-reported. The Ethics Committee of the Helsinki and Uusimaa Hospital District approved the study protocol (327/13/03/00/2015, GDPR update 587/2019). All participants provided web-based written informed consent.

### Measures of obesity

At baseline, participants self-reported height, weight, and waist circumference via an online questionnaire. They were instructed to record weight in the morning after voiding using a home scale. Waist circumference was self-measured at the narrowest point between the lower ribs and iliac crest, aided by a visual guide. Weekly weight reporting was encouraged, with daily reporting available, while waist circumference was reassessed at three-, six-, nine-, and 12-months. Interpolated weights at these timepoints were used to calculate follow-up BMI (kg/m^2^), and percent changes in weight and waist circumference served as outcome measures in the longitudinal repeated-measures analyses. At 12-months, participants were categorized based on achieving clinically significant weight loss (≥5%).

### Alcohol consumption

Alcohol use was included in both the intervention content and repeated lifestyle assessments. It was addressed in relation to energy intake, sleep, appetite and cravings, eating behavior, recovery, and maintenance of lifestyle change. Participants were encouraged to reflect on whether alcohol use affected their weight management and could request additional material on alcohol use. Coaches provided nonjudgemental support and encouraged reduction or cessation of alcohol use when relevant. Alcohol consumption frequency (“How often do you drink beer, wine, or other alcoholic beverages?”) and single-session dose (“How many drinks do you usually have on the days you drink alcohol?”) were assessed at baseline and at three, six, nine, and 12 months using self-reported questionnaires without in-person interviews (alcohol and smoking questions were adapted from established questionnaires [17, 18]). Frequency was reported on a seven-point scale (never; <once/month; ∼once/month; two–three/month; once/week; two–four/week; >four/week) and converted to weekly episodes using prespecified mid-points: 0, 0.125, 0.25, 0.625, 1.0, 3.0, and 5.5 occasions/week (the last two reflect 12 and 22 occasions/month ÷ 4). Single-session dose was defined as the number of standard alcohol units.

For each timepoint, we calculated weekly alcohol intake as:


Weekly units=frequency (occasions/week)×single-session dose

Participants were categorized into three groups based on baseline alcohol consumption: abstinent (self-reportedly not consuming alcohol), low-risk drinking (any alcohol consumption below the threshold for risky drinking), and risky drinking (>14 units/week or >six units/occasion for men, and >seven units/week or >five units/occasion for women). Weekly and single-session doses were used as continuous variables in the analyses. For each follow-up timepoint, we calculated changes from baseline in reported weekly and single-session alcohol doses.

### Smoking

Smoking was included in HWC through repeated lifestyle assessments and broader lifestyle counselling, although it had no dedicated weekly module. Smoking was addressed in relation to stress management, fatigue, and well-being, with additional self-care programs for cessation available. Coaches encouraged smoking cessation when relevant. At baseline, participants indicated smoking status (non-smoker, occasional smoker, or daily smoker). Ever-smokers (i.e. participants reporting any past or current smoking) were additionally asked to specify age at smoking initiation, the number of cigarettes or tobacco products used per day, and, if applicable, the year they quit. Based on provided information, we calculated smoking duration in years, pack-years ([cigarettes per day/20] × years smoked), and categorized individuals into never-smokers, former smokers, and current smokers (occasional and daily smokers).

### Statistical analyses

Data are presented as frequencies (percentages) for categorical variables, medians (interquartile ranges) for continuous variables with skewed distribution, and means ± standard errors for normally distributed variables. Pearson’s chi-squared test was used to investigate group-differences for categorical variables. For continuous variables with skewed distribution, we applied the Mann–Whitney U-test (two groups) and Kruskal–Wallis test (more than two groups), and for normally distributed variables one-way ANOVA (more than two groups). Repeated measures ANOVA was used to examine changes in continuous variables over the 12-month program. Clustering of baseline alcohol and smoking habits (monthly alcohol consumption frequency, weekly alcohol intake, and three-level smoking status) was examined using TwoStep cluster analysis. Using generalized linear models, we investigated associations of baseline alcohol consumption (abstinent [reference], low-risk drinking, and risky drinking; and continuous weekly and single-session alcohol doses) and smoking (three-level smoking status with never smokers as the reference; and smoking duration and pack-years) with baseline obesity measures (BMI and waist circumference). To study associations of alcohol consumption changes (weekly and single-session alcohol doses) by baseline alcohol consumption categories and baseline smoking (three-level smoking status, duration, and pack-years) with the odds of achieving ≥5% weight loss, we used logistic regression analysis. Generalized linear models for repeated measures examined associations of changes in alcohol consumption by baseline alcohol consumption categories and baseline smoking habits with percent changes in weight and waist circumference over 12 months. All multivariable models were adjusted for age and sex. Analyses with alcohol-related predictors were additionally adjusted for smoking status, while analyses with smoking-related predictors were adjusted for baseline alcohol consumption category. Models with percent changes in weight and waist circumference as outcomes were further adjusted for baseline BMI and waist circumference, respectively. Analyses were conducted with IBM SPSS Statistics, version 30.0.0.0 (IBM Corp., Armonk, NY, USA), with significance set at *P* < 0.05. Secondary outcomes were considered exploratory; thus, no correction for multiple testing was performed.

## Results

### Study population

Of the 2157 individuals enrolled in the HWC program, 1719 (79.7%) were included in the analyses after excluding 182 who used additional weight loss methods and 256 with missing smoking and alcohol data (Supplementary Fig. S1). Participants included in the analyses were predominantly women (83.3%) with a mean baseline weight of 110 kg, BMI of 39.1 kg/m^2^, and waist circumference of 118 cm. Among excluded participants, these values were 79.2%, 113 kg, 40.0 kg/m^2^, and 120 cm (Supplementary Table S1).

### Alcohol

#### Baseline alcohol consumption

At baseline, 359 participants (20.9%) reported no alcohol consumption, while 1360 (79.1%) reported alcohol consumption at some frequency: 140 (8.1%) less than once a month, 532 (31.0%) once a month, 349 (20.3%) two to three times a month, 164 (9.5%) once a week, 159 (9.3%) two to four times a week, and 16 (0.9%) more than four times a week (Supplementary Fig. S2). Overall, 1082 individuals (62.9%) were classified as low-risk drinkers and 278 (16.2%) as risky drinkers, with a greater proportion of men reporting risky drinking compared with women (Table 1). The median (interquartile range) weekly alcohol dose was 0.8 (0.4–1.9) units among low-risk drinkers and 6.3 (2.0–12.0) units among risky drinkers. Units per drinking occasion increased with drinking frequency (Supplementary Fig. S3). Abstainers, low-risk drinkers, and risky drinkers differed in several baseline characteristics (Table 1). The risky-drinking group had the highest proportion of men, was youngest on average, and had the greatest weight, waist circumference, BMI, and prevalence of current smoking.

**Table 1.** Baseline characteristics by alcohol consumption categories and smoking status^a^

	Abstinence	Low-risk drinking	Risky drinking		Never smoking	Former smoking	Current smoking	
	*n* = 359 (20.9%)	*n* = 1082 (62.9%)	*n* = 278 (16.2%)	*P*	*n* = 1128 (65.6%)	*n* = 383 (22.3%)	*n* = 208 (12.1%)	*P*
In women, *n* (%)	302 (21.1)	927 (64.7)	203 (14.2)	<0.001	971 (67.8)	298 (20.8)	163 (11.4)	<0.001
In men, *n* (%)	57 (19.9)	155 (54.0)	75 (26.1)		157 (54.7)	85 (29.6)	45 (15.7)	
Age, years	50 (39-58)	52 (43-59)	49 (39-57)	<0.001	51 (42-58)	54 (46-62)	45 (36-55)	<0.001
Weight, kg	111 (99–129)	109 (97–124)	116 (100–130)	<0.001	110 (97–125)	109 (98–125)	114 (100–127)	0.281
Waist, cm	119 (110–129)	117 (108–127)	122 (111–133)	<0.001	117 (108–128)	120 (110–132)	120 (111–130)	0.005
BMI, kg/m^2^	39.7 (35.6–44.8)	38.6 (35.1–43.0)	40.1 (36.0–43.9)	0.002	39.1 (35.0–43.5)	38.6 (35.5–43.4)	39.7 (36.4–43.8)	0.314
Alcohol dose/week	0.0 (0.0–0.0)	0.8 (0.4–1.9)	6.3 (2.0–12.0)	<0.001	0.5 (0.1–1.9)	0.8 (0.3–2.5)	1.9 (0.5–4.5)	<0.001
Current smoking, *n* (%)	24 (6.7)	101 (9.3)	83 (29.9)	<0.001	–	–	–	
Smoking duration, years	4.0 ± 0.6	4.2 ± 0.4	9.5 ± 0.9	<0.001	0 ± 0	23.3 ± 1.2	27.8 ± 0.9	<0.001
Pack-years	1.3 **±** 0.3	1.2 **±** 0.2	3.8 ± 0.7	<0.001	0.0 ± 0.0	12.7 ± 5.0	12.0 ± 0.9	<0.001

a Categorical variables are displayed as frequencies (percentages). Continuous variables with skewed distribution are displayed as medians (interquartile ranges) except for smoking duration and pack-years which are displayed as mean ± standard error. To investigate the group differences in categorical variables, the Pearson’s chi-squared test was applied. For continuous variables, the Kruskal–Wallis test was used. Abstinence, reporting no alcohol consumption at baseline; Low-risk drinking, reporting any alcohol consumption below the threshold for risky drinking; Risky drinking, >14 units/week or >6 units/occasion for men, and >7 units/week or >5 units/occasion for women; Current smoking, self-reportedly smoking daily or occasionally; BMI, body mass index.

#### Baseline alcohol consumption and baseline obesity measures

Adjusted for age, sex, and smoking status, low-risk drinking was associated with a lower baseline BMI compared with abstinence (*B* = −1.307 [95% confidence interval (CI), −2.078 to −0.537]), while a higher single-session dose was linked to greater waist circumference (*B* = 0.420 [95% CI, 0.138–0.701]) (Supplementary Table S2). Risky drinking and weekly alcohol dose were not associated with baseline obesity measures.

#### Change in alcohol consumption over the 12-month program

**Figure 1.**
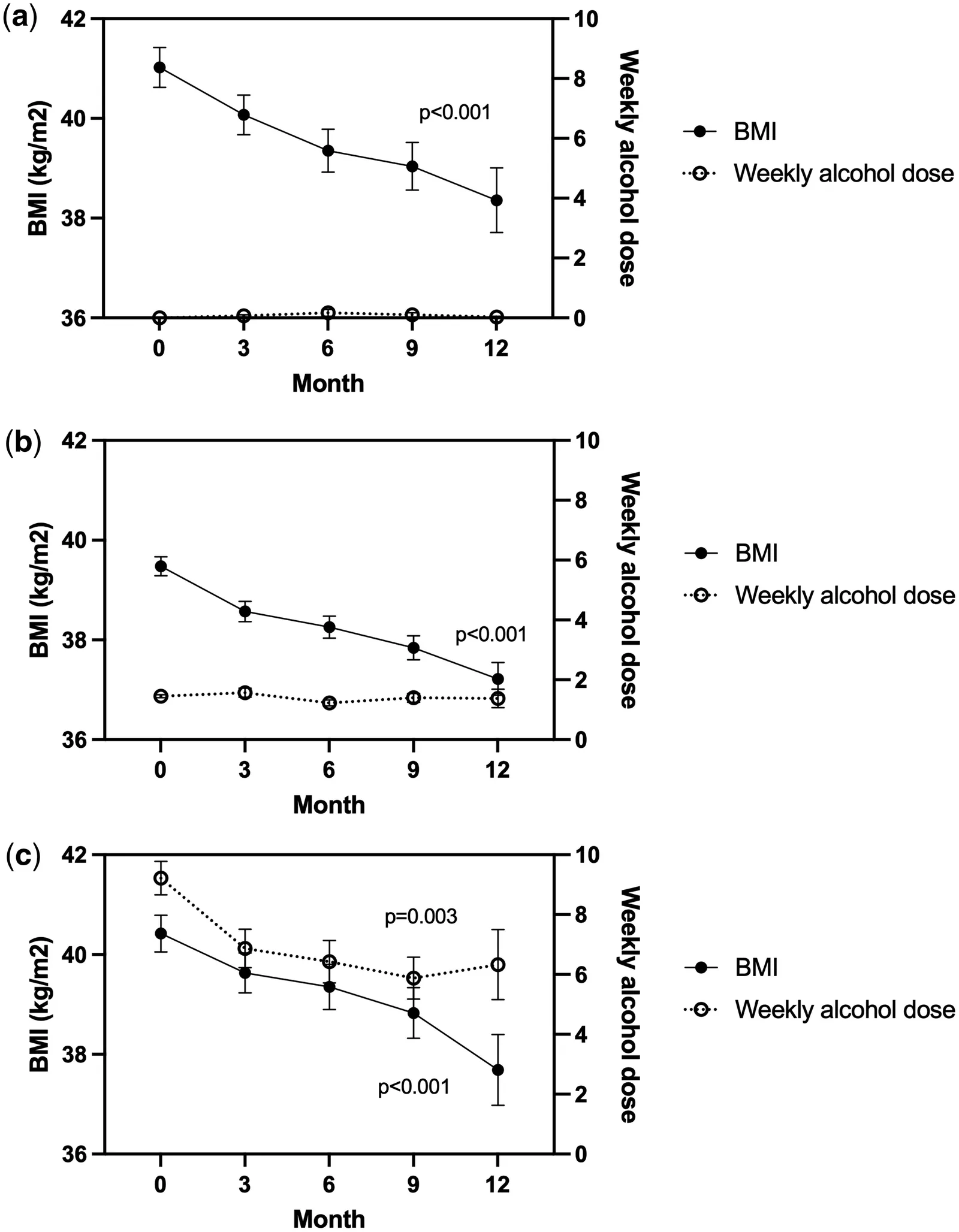
BMI and weekly alcohol dose trajectories across the 12-month program of individuals reporting (a) no alcohol use (b) low-risk alcohol use, and (c) high-risk alcohol use at baseline. Mean (SEM). Repeated measures ANOVA.

Both reported weekly and single-session alcohol doses decreased over the program (Supplementary Fig. S4a and b). The reduction depended on baseline consumption, with a significant decline only among participants reporting risky drinking. Figure 1 illustrates trajectories of weekly alcohol dose and BMI over the program for participants reporting abstinence (a), low-risk drinking (b), and high-risk drinking (c) at baseline. Adjusted for age, sex, and smoking status, a higher baseline weekly alcohol dose predicted a greater reduction in weekly dose (*B* = −0.458 [95% CI, −0.484 to −0.433]). Similarly, a higher baseline single-session dose predicted a greater reduction in single-session dose (*B* = −0.204 [95% CI, −0.229 to −0.179]).

#### Change in alcohol consumption and weight loss outcomes

Among baseline non-drinkers, increased alcohol intake over 12 months was associated with an attenuated reduction in waist circumference, regardless of whether exposure was assessed as weekly or single-session dose (Table 2). In contrast, among participants reporting risky drinking at baseline, cutting alcohol intake was associated with greater weight loss and a larger decrease in waist circumference. Changes in alcohol consumption were not associated with the odds of achieving ≥5% weight loss (Supplementary Table S3).

**Table 2.** Associations between changes in alcohol consumption stratified by baseline alcohol consumption, smoking habits, and weight loss outcomes across a 12-month digital lifestyle intervention, the Healthy Weight Coaching^a^

	Relative weight change	Relative waist circumference change
	*B* (95% Wald confidence interval)	*B* (95% Wald confidence interval)
Change in weekly alcohol dose^b^^,^^d^		
Abstinence	0.417 (−0.057 to 0.891)	0.762 (0.227 to 1.296)
Low-risk drinking	−0.065 (−0.144 to 0.013)	−0.049 (−0.149 to 0.051)
Risky drinking	0.062 (0.005 to 0.119)	−0.019 (−0.107 to 0.070)
Change in single-session dose^b^^,^^d^		
Abstinence	−0.251 (−0.609 to 0.107)	1.168 (0.183 to 2.153)
Low-risk drinking	−0.074 (−0.228 to 0.080)	0.075 (−0.133 to 0.283)
Risky drinking	0.321 (0.141 to 0.502)	0.381 (0.034 to 0.727)
Baseline smoking, smoking history^c^^,^^e^		
Never smoking	Reference group	Reference group
Former smoking	0.334 (−0.001 to 0.670)	0.195 (−0.334 to 0.723)
Current smoking	−0.393 (−0.843 to 0.056)	−0.208 (−0.954 to 0.539)
Smoking duration	−0.002 (−0.015 to 0.011	0.017 (−0.004 to 0.037)
Pack-years	−0.009 (−0.035 to 0.017)	0.059 (0.021 to 0.098)

a Generalized linear model for repeated measures.

b Adjusted for age, sex, smoking status, and baseline measure of obesity (body mass index for relative weight change and waist circumference for relative waist circumference change).

c Adjusted for age, sex, alcohol consumption classification, and baseline measure of obesity (body mass index for relative weight change and waist circumference for relative waist circumference change).

d Abstinence, reporting no alcohol consumption at baseline; Low-risk drinking, reporting any alcohol consumption below the threshold for risky drinking; Risky drinking, >14 units/week or >6 units/occasion for men, and >7 units/week or >5 units/occasion for women.

e Current smoking, self-reportedly smoking daily or occasionally.

### Smoking

#### Smoking habits

Of the participants, 1128 (65.6%) were never-smokers, 383 (22.3%) former smokers, and 208 (12.1%) current smokers (94 [5.5%] daily and 114 [6.6%] occasional) (Table 1 and Supplementary Fig. S5). Never-smokers had the highest proportion of women, whereas current smoking was more common among men and current smokers were youngest on average (Table 1). Alcohol consumption was lowest among never-smokers and highest among current smokers. Mean smoking duration was 23.3 years among former smokers and 27.8 years among current smokers, while mean pack-years were 12.7 and 12.0, respectively.

#### Smoking and baseline obesity measures

Adjusted for age, sex, and alcohol consumption category, smoking variables were not associated with baseline BMI (Supplementary Table S2). In contrast, compared with never-smokers, former smoking was associated with higher waist circumference (*B* = 2.039 [95% CI, 0.256–3.822]). Similarly, longer smoking duration was associated with greater waist circumference (*B* = 0.075 [95% CI, 0.006–0.143]).

#### Smoking and weight loss outcomes across the program

**Figure 2.**
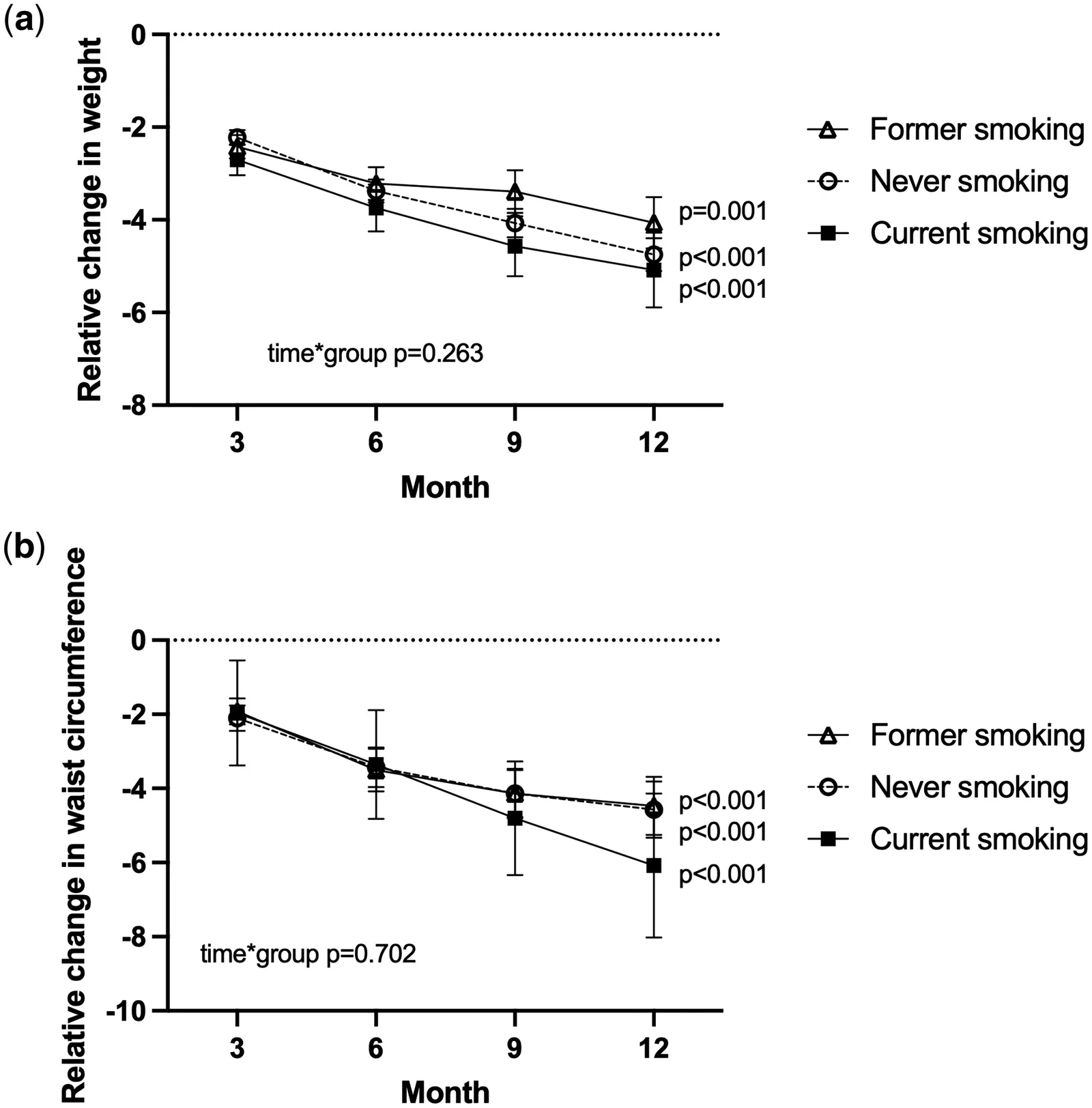
Relative change in (a) weight and (b) waist circumference by the three-level baseline smoking status. Repeated measures ANOVA.

Using never-smokers as reference, neither former nor current smoking was associated with 12-month weight loss outcomes (Table 2). Similarly, smoking duration showed no association with these outcomes. In contrast, a higher number of pack-years was associated with an attenuated reduction in waist circumference. Trajectories of relative changes in weight and waist circumference by smoking status are shown in Fig. 2a and b. All smoking categories showed similar overall trends over time. Smoking habits were not associated with the odds of achieving ≥5% weight loss at 12 months (Supplementary Table S3).

### Alcohol consumption and smoking clusters

Four clusters differing in alcohol and/or smoking habits were identified using TwoStep cluster analysis (Supplementary Table S4): moderate drinkers, non-smokers; heavy drinkers; moderate drinkers, former smokers; and moderate drinkers, current smokers. Heavy drinkers had the lowest proportion of women and the lowest median BMI, whereas moderate drinkers, current smokers were youngest. In adjusted models, compared with moderate drinkers, non-smokers, moderate drinkers, former smokers exhibited less weight loss (*B* = 0.436 [95% CI, 0.077–0.796], Supplementary Table S5).

## Discussion

Using data from a large national sample participating in a digital lifestyle intervention, 79% reported alcohol consumption at some frequency with approximately one in six classified as risky drinkers, a group with the highest crude baseline weight, waist circumference, and body mass index. Alcohol consumption decreased significantly during the program, largely driven by reductions among those with the highest baseline intakes, with the greatest potential for change. Among participants classified as risky drinkers at baseline, reduction in alcohol consumption was associated with improved weight loss outcomes across the 12-month program, while increased alcohol consumption among baseline abstainers was linked to attenuated reduction in waist circumference. Regarding smoking, 22% and 12% of participants were former and current smokers, respectively. Smoking duration and former smoking were associated with greater baseline waist circumference, whereas higher pack-years were linked to smaller waist circumference reductions. Alcohol consumption and smoking also clustered, with moderate drinking combined with former smoking associated with attenuated weight loss. Notably, in the cluster analysis, heavy drinkers included the largest proportion of men, consistent with known sex differences in alcohol consumption patterns [19]. Sex was therefore included as a covariate in all multivariable analyses.

Although alcoholic beverages provide additional energy and may stimulate appetite, epidemiological data paradoxically suggest that light-to-moderate alcohol intake may be protective against obesity [20]. Consistent with this, the lowest median BMI in our study was observed among participants reporting low-risk drinking. However, the difference between abstainers and low-risk drinkers was small and unlikely of clinical relevance.

The association between alcohol consumption and weight loss was also investigated in the Look AHEAD study [10]. Alcohol intake was not associated with weight loss after one year, although participants who remained abstinent over four years lost a greater proportion of their baseline weight than heavy drinkers. Similarly, an observational study of men from general practices in Britain found heavy alcohol consumption to be associated with higher BMI [21]. After five years of follow-up, those who continued to drink heavily showed the greatest weight gain. In contrast, men who were heavy drinkers at baseline but later reduced their intake experienced more weight loss and less weight gain compared with those who continued heavy drinking. These findings may be particularly relevant for the heavy-drinking cluster in our study, which included the largest proportion of men. Kase *et al*. [22] also reported reductions in alcohol intake during a behavioral weight loss program, although, they observed no overall association between reduction in alcohol use and weight change. However, reduced alcohol use was linked to greater weight loss among individuals with higher impulsivity [22], suggesting that alcohol consumption can trigger overeating episodes in susceptible individuals.

Similar to our observations, smoking status was not associated with weight loss outcomes in the Look AHEAD trial [23] or in a systematic review of 48 studies of individuals undergoing bariatric surgery [24]. In contrast, Lundgren *et al*. [13] reported that smoking status was associated with small but meaningful differences in weight loss outcomes, with current smokers consistently losing the least weight. While smoking status itself was not associated with weight loss outcomes in the present study, higher pack-years were linked to smaller reductions in waist circumference, suggesting that cumulative smoking exposure may be more closely related to central adiposity than total weight loss. Interestingly, in the cluster analysis combining alcohol use and smoking habits, individuals classified as moderate drinkers and former smokers exhibited significantly less weight loss compared with moderate drinkers and non-smokers. This finding may reflect the lasting metabolic and behavioral consequences of long-term smoking history. Although nicotine acutely increases resting energy expenditure and suppresses appetite, these effects may be attenuated in individuals with obesity or long-term smoking history [25, 26]. Moreover, chronic smoking has been linked to greater central adiposity despite lower overall body weight possibly through alterations in cortisol regulation, insulin sensitivity, and fat distribution [4]. The observation that former smokers in our sample had the highest cumulative smoking exposure, measured as pack-years, further supports the notion that long-term smoking may leave persistent metabolic effects even after smoking cessation.

Beyond its many well-documented harmful health consequences [27], smoking is often accompanied by other unhealthy behaviors such as lower intake of fruits and vegetables, reduced physical activity, and greater alcohol consumption [28]. Notably, the combination of poor diet and smoking has been linked to elevated risks of stroke, cardiovascular disease, and all-cause mortality [29], highlighting that smoking cessation is valuable for much more than weight outcomes. Of interest, smoking prevalence (12.1%), in the current study, was somewhat lower than that reported in individuals with overweight or obesity in the FINRISK study (18.7%) [30]. This discrepancy may reflect differences in sampling frame, recruitment bias, social desirability in reporting, or secular trends in smoking rates. Regardless, from public health perspective, there is a need for continued efforts to promote smoking cessation, as quitting not only reduces multiple health risks but also complements broader strategies for improving overall well-being.

This study has several strengths. It included a large national sample of real-world patients, which enhances the generalizability of the findings. Data were collected from a digital lifestyle intervention, which is notable because no prior studies have examined the associations between smoking and/or alcohol consumption and weight loss in fully digital programs. Participants were enrolled via physician referral, which may improve clinical relevance. Compared with traditional randomized controlled trials, our sample was likely more diverse, as randomized controlled trials often have restrictive eligibility criteria; this diversity increases the applicability of the findings to routine clinical practice. The 12-month duration further strengthens the study, as it reflects the long-term management required for effective obesity treatment [31]. However, there are also limitations. By design, it was uncontrolled, limiting causal inference. Women were overrepresented compared with the worldwide obesity prevalence by sex (15% female and 11% male) [32], although this imbalance is common in behavioral weight loss studies [33]. Sex was adjusted for in all multivariable analyses, but the smaller number of men limited meaningful sex-specific analyses. All data were self-reported, potentially introducing bias; in particular, alcohol consumption is frequently under-reported [34]. Nevertheless, standardized and weekly weight assessments reduce the risk of gross misreporting, and repeated alcohol measures across the program likely captured relative changes over time. Additionally, online reporting format likely mitigates social desirability bias due to limited direct interaction with coaches. Finally, the lack of follow-up data on smoking prevented us from investigating the changes in smoking habits across the program. Overall, the combination of a large, diverse sample of real-world participants, a longitudinal digital intervention, and robust weight tracking supports the relevance of these findings for clinical practice while acknowledging the inherent limitations of self-reported, observational data.

In conclusion, participation in this digital lifestyle intervention was associated with meaningful reductions in alcohol consumption, particularly among participants reporting risky drinking at baseline. These reductions were reflected in greater weight loss. Higher pack-years were associated with smaller reductions in waist circumference, and being a former smoker combined with moderate drinking attenuated weight loss, suggesting that cumulative smoking exposure may interfere with weight loss efforts. Overall, these observations highlight the value of addressing alcohol consumption and smoking in obesity management, as modifying these behaviors may improve body composition and support broader health outcomes. Behavioral weight loss programs may benefit from incorporating structured counselling on alcohol use and smoking habits, including screening for risky drinking, guidance on moderation, cessation, and post-cessation weight gain.

## Supplementary Material

ckag072_Supplementary_Data

## Data Availability

The participants of this study did not give written consent for their data to be shared publicly, so due to the sensitive nature of the research, supporting data are not available. Key points Reduction in alcohol consumption during the digital lifestyle intervention was observed among participants with risky baseline drinking, and this reduction was associated with greater weight loss. Cumulative smoking exposure, reflected by higher pack-years, was linked to smaller reductions in waist circumference despite similar weight loss across smoking-status groups. Alcohol consumption and smoking were observed to cluster, with moderate drinkers who were former smokers experiencing attenuated weight loss, suggesting lasting metabolic effects of long-term smoking. The findings demonstrate that scalable digital interventions can effectively reduce alcohol consumption, particularly among high-risk drinkers. Public health programs that provide support in reducing alcohol consumption and smoking could be combined with obesity management programs, as targeting these modifiable behaviors may improve weight-loss outcomes.
