# Supplementary material for: Alcohol consumption, smoking, and weight loss outcomes: findings from a 12-month digital lifestyle intervention
Source: Eur J Public Health. 2026 May 19;36(3):ckag072. doi: 10.1093/eurpub/ckag072 (PMC13186192; doi:10.1093/eurpub/ckag072)
Supplement: ckag072_Supplementary_Data [file ckag072_supplementary_data.pdf]

Alcohol consumption, smoking, and weight loss outcomes: Findings from a 12-month digital lifestyle intervention

Emma R. Wu[1], Anu Joki[2,3], Mikko S. Venäläinen[4], Laura-Unnukka Suojanen[3], Kirsi H. Pietiläinen[2,3†*], Aila J. Ahola[2,3†]

[1] University of South Florida Morsani College of Medicine, Tampa, FL, USA

[2] Obesity Research Unit, Research Program for Clinical and Molecular Metabolism, Faculty of Medicine, University of Helsinki, Helsinki, Finland

[3] Healthy Weight Hub, Abdominal Centre, Helsinki University Hospital and University of Helsinki, Helsinki, Finland

[4] Department of Medical Physics, Turku University Hospital and University of Turku, Turku, Finland

[†] Shared authorship

*Corresponding author Kirsi Pietiläinen, Obesity Research Unit, Biomedicum 1, Haartmaninkatu 8, PO Box 63, FI-00014 University of Helsinki, Finland. Tel +358 505992295. Email: kirsi.pietilainen@helsinki.fi

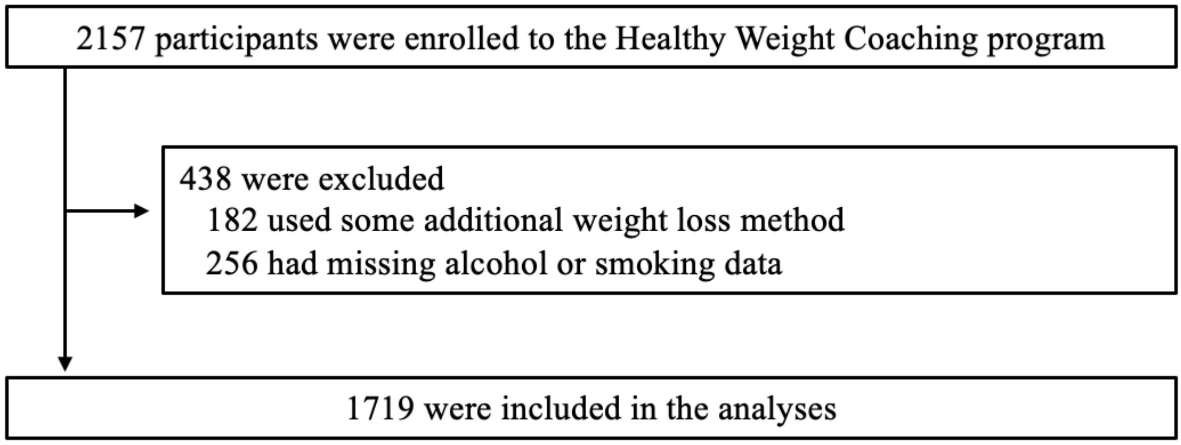

Supplementary Figure 1 Flowchart of participants included and excluded from the study population

Supplementary Table 1 Baseline characteristics of included vs. excluded populations

	**Included**	**Excluded**
	n=1719 (79.7%)	n=438 (20.3%)
Women, n (%)	1432 (83.3)	347 (79.2)
Age, years	51 (42-59)	53 (43-59)
Weight, kg	110 (98-125)	113 (99-131)
Body mass index, kg/m^2	39.1 (35.3-43.5)	40.0 (36.7-44.6)
Waist circumference, cm	118 (109-129)	120 (111-133)

Data are presented as frequency (percentage) for the categorical variable and medians (interquartile ranges) for continuous variables.

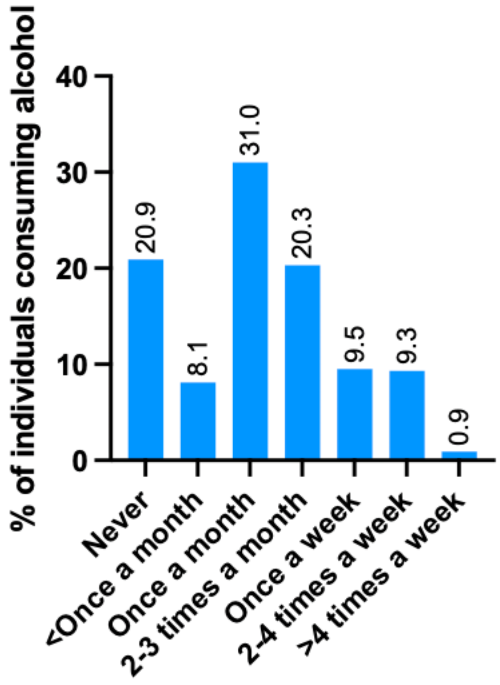

Supplementary Figure 2 Percentage of individuals (n=1719) reporting various frequencies of alcohol consumption at baseline

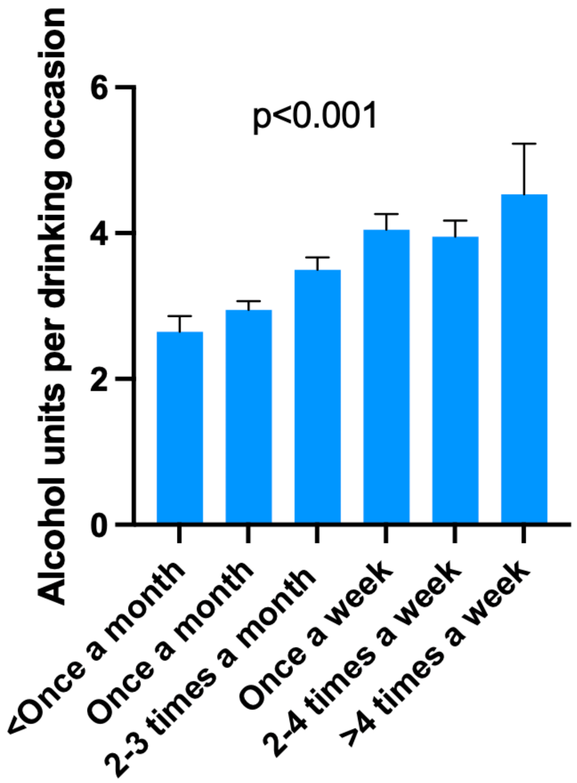

Supplementary Figure 3 Mean number of alcohol units per drinking occasion by frequencies of drinking at baseline. One-way ANOVA $p<0.001$

Supplementary Table 2 Associations between baseline alcohol consumption, smoking habits, and baseline measures of obesity

	**Body mass index**		**Waist circumference**	
	B (95% Wald confidence interval)	EMM ± SE	B (95% Wald confidence interval)	EMM ± SE
Alcohol[1]				
Abstinence	Reference group	40.9 ± 0.3	Reference group	120 ± 0.8
Low-risk drinking	-1. 307 (-2.078 – -0.537)	39.6 ± 0.2	-0.951 (-2.762 – 0.860)	119 ± 0.5
Risky drinking	-0.812 (-1.847 – 0.223)	40.1 ± 0.4	1.360 (-1.122 – 3.841)	121 ± 1.0
Weekly dose	-0.029 (-0.092 – 0.035)		0.073 (-0.074 – 0.220)	
Single-session dose	0.047 (-0.067 – 0.162)		0.420 (0.138 – 0.701)	
Smoking[2]				
Never smoking	Reference group	39.9 ± 0.2	Reference group	119 ± 0.5
Former smoking	0.292 (-0.465 – 1.050)	40.2 ± 0.3	2.039 (0.256 – 3.822)	121 ± 0.8
Current smoking	0.114 (-0.867 – 1.096)	40.0 ± 0.5	0.731 (-1.599 – 3.061)	119 ± 1.1
Smoking duration	0.013 (-0.018 – 0.043)		0.075 (0.006 – 0.143)	
Pack-years	0.017 (-0.041 – 0.075)		0.089 (-0.042 – 0.221)	

Generalized linear model. [1]Adjusted for age, sex, and smoking status; [2]adjusted for age, sex, and alcohol consumption classification. EMM, estimated marginal mean; SE, standard error; Abstinence, reporting no alcohol consumption at baseline; Low-risk drinking, reporting any alcohol consumption below the threshold for risky drinking; Risky drinking, >14 units/week or >6 units/occasion for men, and >7 units/week or >5 units/occasion for women; Current smoking, self-reportedly smoking daily or occasionally.

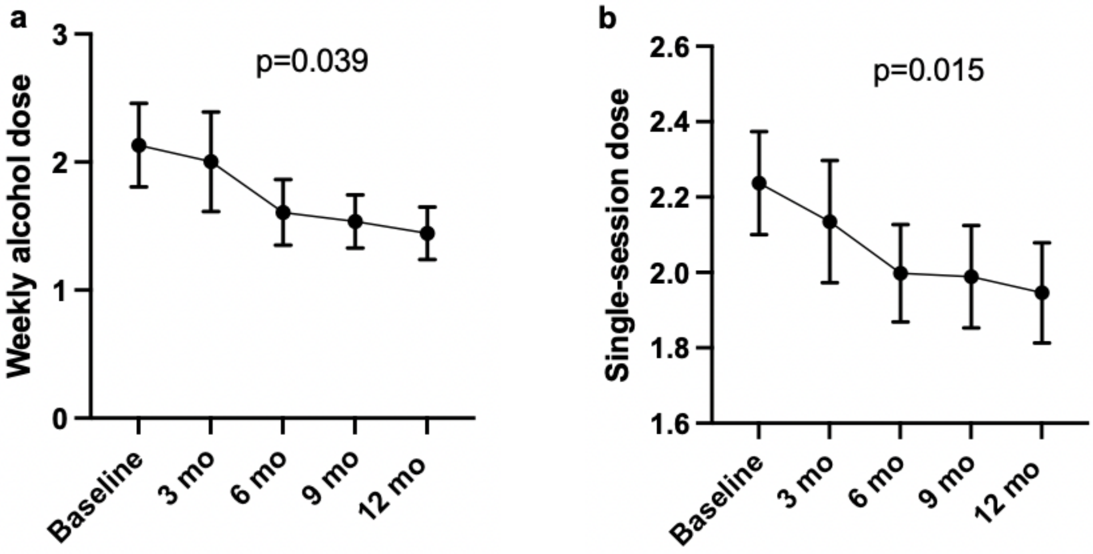

Supplementary Figure 4 Changes in **a)** weekly alcohol dose and **b)** single-session dose across the 12-month Healthy Weight Coaching program. Data are shown as mean (SEM). Repeated measures ANOVA.

Supplementary Table 3 Associations between changes in alcohol consumption stratified by baseline alcohol consumption, smoking habits, and the odds of achieving ≥5% weight loss

	**≥5% weight loss**
	Odds ratio (95% confidence interval)
Change in weekly alcohol dose[1]	
Abstinence	0.686 (0.131 – 3.595)
Low-risk drinking	1.030 (0.973 – 1.091)
Risky drinking	0.965 (0.928 – 1.003)
Change in single-session dose[1]	
Abstinence	1.081 (0.833 – 1.403)
Low-risk drinking	1.010 (0.915 – 1.115)
Risky drinking	0.874 (0.759 – 1.006)
Baseline smoking, smoking history[2]	
Never smoking	Reference group
Former smoking	0.737 (0.480 – 1.132)
Current smoking	1.390 (0.750 – 2.277)
Smoking duration	0.994 (0.978 – 1.009)
Pack-years	0.991 (0.955 – 1.029)

Logistic regression. [1]Adjusted for age, sex, smoking status, and baseline body mass index; [2]Adjusted for age, sex, alcohol consumption classification, and baseline body mass index. Abstinence, reporting no alcohol consumption at baseline; Low-risk drinking, reporting any alcohol consumption below the threshold for risky drinking; Risky drinking, >14 units/week or >6 units/occasion for men, and >7 units/week or >5 units/occasion for women; Current smoking, self-reportedly smoking daily or occasionally.

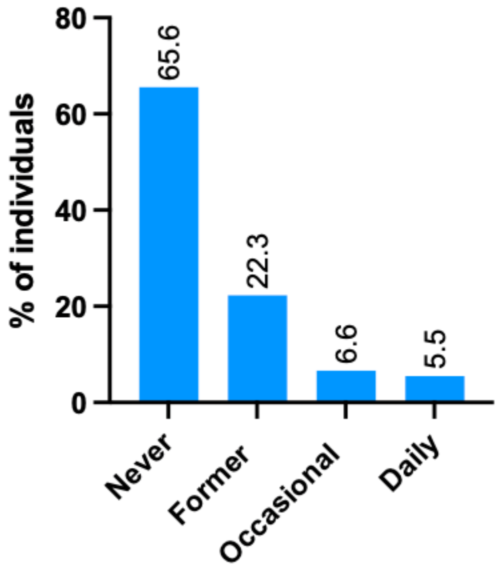

Supplementary Figure 5 Percentage of individuals (n=1719) reporting smoking habits at baseline

Supplementary Table 4 TwoStep cluster analysis-derived alcohol consumption and smoking clusters

	Moderate drinkers, non-smokers	Heavy drinkers	Moderate drinkers, former smokers	Moderate drinkers, current smokers	
	N=1010 (59.5%)	N=173 (10.2%)	N=332 (19.6%)	N=183 (10.8%)	p
Alcohol frequency/month	1.0 (0 – 2.5)	12.0 (12.0 – 12.0)	1.0 (0.5 – 2.5)	1.0 (1.0 – 2.5)	**<0.001**
Alcohol dose/week	0.5 (0 – 1.3)	10.5 (6.0 – 18.0)	0.5 (0.1 – 1.9)	1.5 (0.4 – 3.1)	**<0.001**
Smoking status, %					**<0.001**
Never	100	63.6	0	0	
Former	0	23.1	100	0	
Current	0	13.3	0	100	
Women, n (%)	881 (87.2)	117 (67.6)	264 (79.5)	150 (82.0)	**<0.001**
Age, years	55 (40 – 58)	55 (48 – 61)	54 (45 – 61)	44 (35 – 55)	**<0.001**
Weight, kg	111 (97 – 125)	110 (96 – 125)	109 (98 – 125)	115 (100 – 128)	0.428
Body mass index, kg/m^2	39.2 (35.2 – 43.7)	37.2 (33.8 – 42.0)	38.6 (35.6 – 43.4)	39.8 (36.5 – 43.9)	**0.003**
Waist circumference, cm	117 (108 – 127)	120 (110 – 131)	120 (110 – 130)	120 (111 – 130)	**0.031**

Data are presented as frequencies (percentages) for the categorical variables and medians (interquartile ranges) for continuous variables. The chi-squared test was conducted for the categorical variables and the Kruskal-Wallis test for the continuous variables. Monthly alcohol consumption frequency, weekly alcohol dose, and smoking status were included as variables in the cluster analysis. Current smoking, self-reportedly smoking daily or occasionally.

Supplementary Table 5 Associations between TwoStep cluster analysis-derived alcohol and smoking clusters and weight loss outcomes across the 12-month Healthy Weight Coaching program

**Clusters**	**Relative weight change**	**Relative waist circumference change**
	B (95% Wald confidence interval), p	B (95% Wald confidence interval), p
Moderate drinkers, non-smokers	Reference group	Reference group
Heavy drinkers	-0.210 (-0.670 – 0.250), 0.371	-0.181 (-0.893 – 0.530), 0.617
Moderate drinkers, former smokers	**0.436 (0.077 – 0.796), 0.017**	-0.114 (-0.685 – 0.458), 0.697
Moderate drinkers, current smokers	-0.394 (-0.873 – 0.085), 0.107	-0.205 (-1.008 – 0.597), 0.616

Generalized linear model for repeated measures. Adjusted for age, sex, and baseline measure of obesity (body mass index for relative weight change and waist circumference for relative waist circumference change).
